# Hypertrophy of the Nose; Cure

**Published:** 1854-10

**Authors:** 

**Affiliations:** Liverpool


*Hypertrophy of the Nose; Cure.* By Mr. Bickersteth, of Liverpool.—One of these shocking cases of deformity came under my care in June, 1853. The subject was a fine hale man, aged 68, who said his nose had been the ruin of him, and begged I would "cut it off altogether, for he could stand it no longer." It was certainly of a very large size, covering a considerable portion of both cheeks, and hanging over his mouth, so that he could not eat or drink without first lifting it out of the way with his hand. The poor fellow had formerly been in a merchant's office, but was dismissed nearly twelve years ago as his nose was then beginning to assume a disreputable appearance. Having saved some money, he opened a public house, trusting his customers would be less fastidious than his late employers, and for a few years his affairs prospered; but at length, as his nose got larger and larger, his shop was gradually deserted, and he was obliged to give it up. Although a very sober man, he was regarded as a warning of the consequences of free drinking, and his oldest customers preferred to spend their time elsewhere. After this he tried to get all sorts of employment, but without success. One look at him was enough. His nose condemned him. The children cried after him wherever he went. Broken-hearted and disgusted, he lately remained altogether indoors, existing on the charity of his neighbours. The day before I saw him he had ventured out a short distance from home, but was "hooted at" by the children, and was obliged to retreat. In a fit of desperation, he then "determined to have his nose cut off, even if it should cost him his life."

The tumor consisted of a blue-colored mass as large as a closed fist, growing from the point and both alæ of the nose. It was divided into three principal lobes, and each consisted of numerous smaller lobules, which were rough and marked with deep pits and eminences, the orifices of sebaceous ducts.

He was admitted to the Erskine-street Hospital, and on the 15th of June I cut the whole of the tumor off with a bistoury, taking care, with my fingers in the nostrils, to go as near as possible to them without wounding the cartilages of the nose. Afterwards I clipped off with a pair of curved scissors a few projections that remained, and made both sides as symmetrical as possible. The bleeding was rather considerable at the time, but soon stopped under the influence of cold and pressure. Three or four of the larger vessels were secured by ligature.

In the course of three or four days suppuration was established, and the dressings being removed, zinc lotion was applied over the raw surface. Cicatrization quickly took place, and in a fortnight he left the hospital with wonderfully little deformity.

A year has now elapsed, and there is no appearance of a return of the disease. The nose is perfect in its shape, and the new cuticle covering it is quite as smooth as the skin on any part of the face. But for previous knowledge, it would be difficult to suppose anything had been the matter with his nose. With the loss of the deformity, the man has regained his character, and has now no trouble in obtaining constant occupation.—*Monthly Jour. of Med. Sci.*

